# Supplementary material for: Neural energy coding patterns of dopaminergic neural microcircuit and its impairment in major depressive disorder: A computational study
Source: PLoS Comput Biol. 2025 Apr 7;21(4):e1012961. doi: 10.1371/journal.pcbi.1012961 (PMC12002636; doi:10.1371/journal.pcbi.1012961)
Supplement: S1 File — (PDF) [file pcbi.1012961.s001.pdf]

Neural energy coding patterns of dopaminergic neural microcircuit and its impairment in major depressive disorder: A computational study

Supplementary File 1

Yuanxi Li[1, 5*], Bing Zhang[2, 3, 5], Jinqi Liu[4], Rubin Wang[1*]

[1]Institute for Cognitive Neurodynamics, School of Mathematics, East China University of Science and Technology, Shanghai, 200237, China.
[2]Department of Anesthesiology, Obstetrics and Gynecology Hospital of Fudan University, Fudan University, Shanghai, 200082, China.
[3]Shanghai Key Laboratory of Maternal Fetal Medicine, Shanghai Institute of Maternal-Fetal Medicine and Gynecologic Oncology, Department of Anesthesiology, Clinical and Translational Research Center, Shanghai First Maternity and Infant Hospital, Tongji University School of Medicine, Shanghai, 201204, China.
[4]University of Rochester, Rochester, 14627, NY, USA.
[5]These authors contributed equally to the manuscript.

*Corresponding author(s). E-mail(s): dr.yuanxli@gmail.com; rbwang@ecust.edu.cn;
Contributing authors: bingozzz@126.com; jliu161@u.rochester.edu;

1 Structure of the VTA-NAc-mPFC microcircuit

We modeled three key brain regions of the dopaminergic neural circuit in this paper based on the anatomic experiments [1] and our previous papers [2, 3], which were the ventral tegmental area (VTA), the nucleus accumbens (NAc), and the medial prefrontal cortex (mPFC) (See **Figure 1a** in the manuscript). This biological neural network model consisted of 28 neurons. Classified by brain regions, the NAc region consisted of one medium spiny neuron (MSN, D2-type), one parvalbumin interneuron (PV interneuron), and one calbindin interneuron (CB interneuron); The mPFC region consisted of twenty pyramidal neurons (Pyra neurons, D1-type), three PV interneurons, and two CB interneurons; Dopaminergic (DA) neurons in the VTA region were not modeled in this paper because experimental evidence was insufficient to support the development of a computational model, but we set up different dopamine concentration gradients to reflect the neural activity of DA neurons and the dopamine input to the other regions.

Each neuronal type had its unique morphological structure in our model. The MSN in the NAc region consisted of a soma and 10 dendrites with identical ion channel and morphological properties (See **Figure 1b** in the manuscript). The Pyra neurons in the mPFC region included a soma, a proximal dendrite and a distal dendrite (**Fig. 1c** in main text). The PV and CB interneurons were simplified as the structure of only one soma.

2 Neurodynamical modeling based on the H-H model

We used the Hodgkin-Huxley (H-H) model to simulate the membrane potential for each individual neuron, where different types of neurons were considered as different electronic circuit structures. The schematic of H-H model was shown in **Figure 1e** in the manuscript. Basically, the currents of the H-H models in our paper consisted of four main types [2, 3], such as the ion channel currents $\mathrm{I_{Ions}}$, the compartment currents $\mathrm{I_{Compartments}}$, the synaptic currents $\mathrm{I_{Synapses}}$, and the stimuli currents $\mathrm{I_{Stimuli}}$. Then, the H-H model can be described as Equation (S-1).

$$\mathrm{C_m}\frac{\mathrm{dV_m}}{\mathrm{dt}} + \sum \mathrm{I_{Ions}} + \sum \mathrm{I_{Compartments}} + \sum \mathrm{I_{Synapses}} = \mathrm{I_{Stimuli}} \tag{S-1}$$

Where, $\mathrm{C_m} = 1\ \mu\mathrm{F/cm^2}$. $\mathrm{V_m}$ was the membrane potential. t was the simulation time.

There were two types of the ion channel currents in our model, and they were calculated in two different ways. One type was for the currents including most of the $\mathrm{Na^+}$ subtypes, $\mathrm{K^+}$ subtypes, and $\mathrm{Cl^-}$ subtypes. The calculation can be described as Equation (S-2).

$$\begin{cases} \mathrm{I_{Ions}} = \bar{\mathrm{g}}_{\mathrm{Ions}}\mathrm{m^M h^H}(\mathrm{V_m} - \mathrm{E_{Ions}}) \\ \frac{\mathrm{dh}}{\mathrm{dt}} = (\mathrm{h_\infty} - \mathrm{h})/\tau_\mathrm{h} \\ \mathrm{h_\infty}(\mathrm{V_m}) = \frac{1}{1+\exp[(\mathrm{V_m} - \mathrm{V_{1/2,h}})/\mathrm{h_k}]} \end{cases} \tag{S-2}$$

Where, m and h were different types of the voltage gates, $\mathrm{V_{1/2,h}}$ and $\mathrm{h_k}$ came from the fit results of the activation/deactivation curve based on the Boltzmann function: $\mathrm{V_{1/2,h}}$ was the half-activation voltage of the h gate, and $\mathrm{h_k}$ was the slope. The calculation of m gate was similar to h gate.

Some ion channel subtypes, such as $\mathrm{K^+}$ subtypes KAs (slow A-type) channel and KRP (4-AP resistant persistent) channel on the MSN, were described as partially inactivating channels. To calculate it, the first row in Equation (S-2) were modified

as Equation (S-3), where variable 'a' was set up to between 0 and 1, reflecting no inactivation and fully inactivation, respectively.

$$\mathrm{I_{Ions}} = \mathrm{\overline{g}_{Ions} m^M [ah + (1 - a)](V_m - E_{Ions})} \tag{S-3}$$

The other type of currents included most of the Ca^{2+} subtypes. These currents were not only related to the membrane potentials, but also to the concentration and permeability of calcium ions inside and outside the cell membrane, which can be calculated by Equation (S-4) based on the Goldman-Hodgkin-Katz (GHK) equation [4–6].

$$\mathrm{I_{Ca^{2+}}} = \mathrm{P_{Ca^{2+}} z^2 \frac{V_m F^2}{RT} \frac{[Ca^{2+}]_{in} - [Ca^{2+}]_{out} exp(-zFV_m/RT)}{1 - exp(-zFV_m/RT)}} \tag{S-4}$$

Where, $\mathrm{z = 2}$ represented the valence of Ca^{2+} , $\mathrm{F = 96,489\ C/mol}$ represented the Faraday constant, and $\mathrm{R = 8.31\ J/(mol \cdot K)}$ represented the gas constant. $\mathrm{T = 35^{\circ}C}$ was set up according to the experiments [5]. $\mathrm{P_{Ca^{2+}}}$ was introduced to model the permeability of the Ca^{2+} , which can be calculated by Equation (S-5).

$$\mathrm{P_{Ca^{2+}}} = \mathrm{\overline{P}_{Ca^{2+}} m^M h^H} \tag{S-5}$$

The $[Ca^{2+}]_{in}$ and $[Ca^{2+}]_{out}$ in Equation (S-4) represented the extracellular and intracellular Ca^{2+} concentration, respectively, which can be calculated by Equation (S-6). $\mathrm{I_{Ca^{2+}}}$ was the currents of the Ca^{2+} channels. $\mathrm{d} = 0.1\ \mu\mathrm{m}$. The parameters of Ca^{2+} pump were $\mathrm{K_t = 10^{-4}\ mM/ms}$ and $\mathrm{K_d = 10^{-4}\ mM}$. The parameters related to diffusion were $\tau_\mathrm{R} = 43$ ms, $\mathrm{[Ca^{2+}]_{in,inf} = 10^{-5}\ mM}$, $\mathrm{k = 10,000}$, and $\mathrm{p = 0.02}$. See details in REFs [2, 3, 5].

$$\mathrm{\frac{d[Ca^{2+}]_{in}}{dt} = k\frac{-I_{Ca^{2+}}}{2Fd} - p\frac{K_t[Ca^{2+}]_{in}}{[Ca^{2+}]_{in} + K_d} + \frac{[Ca^{2+}]_{in,inf} - [Ca^{2+}]_{in}}{\tau_R}} \tag{S-6}$$

Note that in the above modeling of ion channel dynamics, only voltage-gated ion channels were considered, while other types of ion channels such as mechanosensitive ion channels, temperature-sensitive ion channels, and acid-sensing ion channels were not included due to their complexity.

The compartment currents $\mathrm{I_{Compartments}}$ in Equation (S-1) modeled the the transmission process of neuronal electrical signals over dendrites and axons, especially the passive membrane property such as the dendritic integration process. We considered the substructures (e.g., soma, dendrites, and axons) of a neuron as different cylindrical compartments with ion channels located on their sides. If there was a difference in membrane potential between neighboring compartments, it could cause a current driven by this potential gradient according to the Rall's cable model [7–10], which was described here as the 'compartment current'. As an example, since the soma of MSN was connected to ten identical dendrites, the soma will have 'compartment currents' caused by the ten dendrites. The conductance of the compartment current caused by one dendrite can be described as Equation (S-7).

$$\mathrm{g_{Compartment,MSN,Soma}} = \mathrm{\frac{d_{MSN,Soma} \cdot d^2_{MSN,Dendrite}}{r_L \cdot l_{MSN,Soma} \cdot (d^2_{MSN,Soma} \cdot l_{MSN,Dendrite} + d^2_{MSN,Dendrite} \cdot l_{MSN,Soma})}} \tag{S-7}$$

Where, $\mathrm{r_L}$ denoted the membrane resistance per unit length. The variables 'd' and 'l' were the diameter and length of the cylindrical compartments. Since the soma

of MSN had ten dendrites, its compartment currents can be calculated by Equation (S-8), while other compartment currents can be calculated similarly.

$$I_{Compartment,MSN,Soma} = 10 \times g_{Compartment,MSN,Soma} \cdot (V_{NAc,\ MSN,\ Dendrite} - V_{NAc,\ MSN,\ Soma}) \tag{S-8}$$

The synaptic currents $I_{Synapses}$ in Equation (S-1) in our paper included excitatory postsynaptic currents (EPSCs) related to glutamatergic receptors AMPAR and NMDAR, and inhibitory postsynaptic currents (IPSCs) related to $GABA_aR$. All of them were ligand-gated receptors. The AMPA and $GABA_a$ synaptic currents can be calculated by Equation (S-9) based on Destexhe *et al.* [11].

$$I_{AMPA/GABA_a} = g_{AMPA/GABA_a} \cdot r \cdot (V_m - E_{AMPA/GABA_a}) \tag{S-9}$$

Where, $g_{AMPA/GABA_a}$ denoted the maximum conductance of the synaptic currents, and $E_{AMPA/GABA_a}$ denoted their reversal potentials. r denoted the proportion of the gates, which can be calculated by Equation (S-10).

$$\frac{dr}{dt} = \alpha[T](1 - r) - \beta r \tag{S-10}$$

For the NMDA current, since it was subjected to the block of the magnesium ions, $B(V_m)$ was added to the model so as to match this physiological property [11] as shown in Equation (S-11).

$$\begin{cases} I_{NMDA} = g_{NMDA} \cdot r \cdot B(V_m) \cdot (V_m - E_{NMDA}) \\ B(V_m) = \frac{1}{1+\exp(-(V_m+15)/16.3)} \end{cases} \tag{S-11}$$

The synaptic currents were also related to the strength of network connections. In our VTA-NAc-mPFC neural microcircuit (See **Figure 1a** in the manuscript), the connection strength was described by a matrix $W = \{\omega_{ij} | i, j = 1, 2, ..., 28\}$, where the elements on the diagonal of this matrix were all set to 0, as self-connected synapses in neurons were not considered here. For any pair of neurons, where a_i denoted the presynaptic neuron and a_j denoted the postsynaptic neuron, if they did not have anatomic synaptic connections (e.g., a neuron pair consisting of an interneuron in the NAc region and an interneuron in the mPFC region), the connection strength was set to $\omega_{ij} = 0$; otherwise, if having anatomic synaptic connections (e.g., a neuron pair consisting of a Pyra neuron and an interneuron, both in the mPFC region), the connection strength obeyed the [0,1] uniform distribution. Additionally, the connection strength matrix W was generally not a symmetric matrix, i.e., if a_i and a_j both had synaptic projections to each other, ω_{ij} was usually not equal to ω_{ji}. Then, for any postsynaptic neuron a_j, if its presynaptic neurons were denoted as $a_{i_1}, a_{i_2}, a_{i_3}, ...$, the total synaptic current received by a_j can be calculated by Equation (S-12).

$$I_{Synapses,a_j} = \sum_{k=1,2,3,...} \omega_{i_k,j} \cdot I_{Synapse,a_{i_k} \to a_j} \tag{S-12}$$

The $I_{Stimuli}$ in Equation (S-1) were introduced to mimic the stimuli currents. We usually introduce this term to mimic the stimuli currents in patch clamp electrophysiological experiments *in vitro*.

3 Dopamine input

Different dopamine concentrations (0∼100%) were added to the model in order to mimic the dynamics of the VTA-NAc-mPFC neural microcircuit in normal control group (NC) and major depressive disorder group (MDD). According to Durstewitz *et al.* [12], we considered that different dopamine concentrations linearly changed the some parameters: If one kinetic parameter η was taken to be η_0 with 0% dopamine

input and η_1 with 100% (full) dopamine input, then, for any dopamine concentration 'DARatio' ($0 \le \text{DARatio} \le 1$), $\eta_{\text{DARatio}} = \eta_0 + (\eta_1 - \eta_0) \times \text{DARatio}$.

Not all the ion channel kinetics were affected by dopamine inputs. In Pyra neurons of the mPFC region, the maximum conductance of calcium channels (Ca and Can channels) of soma and distal dendrite, the maximum conductance of slowly inactivating potassium channel (KS channel) and the activating and deactivating parameters of persistent sodium channel (NaP channel) of proximal dendrite were affected by the dopamine input. In the MSN of the NAc region, the maximum conductance of the slowly A-type potassium channel (KAs channel) and Cav1.2 calcium channel (HVA L-type, CaL1.2 channel) of the soma and dendrites were affected by the dopamine input. Note that the model did not study the synaptic plasticity as well as the effects of how dopamine concentration affected the kinetics of AMPA, NMDA, and GABA_a, since there was no critical evidence for their changing kinetic patterns.

4 Detailed parameter settings

Our VTA-NAc-mPFC neural microcircuit model [2, 3] contained 1 MSN (including one soma and ten identical dendrites, 2 different H-H equations in total), 7 interneurons (4 PV interneurons, 3 CB interneurons, only modeling their somata, 7 different H-H equations in total), and 20 Pyra neurons (including one soma, one proximal dendrite, and one distal dendrite for an individual Pyra neuron, 60 different H-H equations in total). Therefore, our model consisted of 69 H-H equations in the form of Equation (S-1), which was a set of ordinary differential equations (ODEs) with 1,527 variables to be solved. Since the ion channel kinetics and the neurotransmitter-receptor binding kinetics were diverse, the neural microcircuit had complex dynamics. The detailed parameters were described in this section.

4.1 Stimuli currents

In our model, the stimulus current $\text{I}_{\text{Stimuli}}$ was set to be uniformly distributed within a certain interval, which were shown in Table (S1).

Table S1 The stimuli currents parameter settings in VTA-NAc-mPFC model [2].

**Neuronal Type**	**Location**	**Stimulus Current** $\mu\text{A} \cdot (\text{cm})^{-2}$
NAc, MSN	Soma	$\text{I}_{\text{Stimuli}} \sim \text{U}[2, 4]$
NAc, MSN	Dendrite	$\text{I}_{\text{Stimuli}} \sim \text{U}[2, 4]$
NAc, PV	-	$\text{I}_{\text{Stimuli}} \sim \text{U}[0.5, 1.5]$
NAc, CB	-	$\text{I}_{\text{Stimuli}} \sim \text{U}[0.1, 0.5]$
mPFC, Pyra	Soma	$\text{I}_{\text{Stimuli}} \sim \text{U}[1.5, 2.5]$
mPFC, Pyra	Proximal Dendrite	$\text{I}_{\text{Stimuli}} \sim \text{U}[1.5, 2.5]$
mPFC, Pyra	Distal Dendrite	$\text{I}_{\text{Stimuli}} \sim \text{U}[1.5, 2.5]$
NAc, PV	-	$\text{I}_{\text{Stimuli}} \sim \text{U}[0.5, 1.5]$
NAc, CB	-	$\text{I}_{\text{Stimuli}} \sim \text{U}[0.1, 0.5]$

4.2 Synaptic currents

The maximum conductances of different synaptic currents in our paper were set up according to the whole-cell electrophysiological experiments and neurodynamic models [5, 12, 13]. The parameters were shown in Table (S2), where the calculations of AMPA and GABA_a postsynaptic currents were based on Equations (S-9, S-10), and the calculations of NMDA postsynaptic currents were based on Equations (S-10, S-11).

Table S2 The parameter settings of synaptic currents in VTA-NAc-mPFC model [2].

Label	Synaptic Type	Presynaptic Neuron	Postsynaptic Neuron	Maximum Conductance mS/cm^2	α	β	Reversal Potential mV
**1**	AMPA	mPFC, Pyra (Soma)	mPFC, PV	3.125e-3	1	0.2	0
**2**	NMDA	mPFC, Pyra (Soma)	mPFC, PV	3.125e-3	7.2e-2	6.7e-3	0
**3**	$GABA_a$	mPFC, Interneurons (PV,CB)	mPFC, PV	7.5e-2	5	0.18	-80
**4**	AMPA	mPFC, Pyra (Soma)	mPFC, CB	3.125e-4	1	0.2	0
**5**	NMDA	mPFC, Pyra (Soma)	mPFC, CB	3.125e-4	7.2e-2	6.7e-3	0
**6**	$GABA_a$	mPFC, Interneurons (PV,CB)	mPFC, CB	7.5e-2	5	0.18	-80
**7**	AMPA	mPFC, Pyra (Soma)	mPFC, Pyra (Distal Dendrite)	1.3e-2	1	0.2	0
**8**	NMDA	mPFC, Pyra (Soma)	mPFC, Pyra (Distal Dendrite)	1.3e-2	7.2e-2	6.7e-3	0
**9**	$GABA_a$	mPFC, Interneurons (PV, CB)	mPFC, Pyra (Soma)	0.1	5	0.18	-80
**10**	AMPA	mPFC, Pyra (Soma)	NAc, MSN (Dendrite)	2e-2	1	0.2	0
**11**	NMDA	mPFC, Pyra (Soma)	NAc, MSN (Dendrite)	2e-2	7.2e-2	6.7e-3	0
**12**	$GABA_a$	NAc, Interneurons (PV, CB)	NAc, MSN (Soma)	0.3	5	0.18	-80
**13**	AMPA	mPFC, Pyra (Soma)	NAc, PV	3.125e-3	1	0.2	0
**14**	NMDA	mPFC, Pyra (Soma)	NAc, PV	3.125e-3	7.2e-2	6.7e-3	0
**15**	$GABA_a$	NAc,CB	NAc,PV	0.3	5	0.18	-80
**16**	AMPA	mPFC, Pyra (Soma)	NAc, CB	3.125e-4	1	0.2	0
**17**	NMDA	mPFC, Pyra (Soma)	NAc, CB	3.125e-4	7.2e-2	6.7e-3	0
**18**	$GABA_a$	NAc, PV	NAc, CB	3e-2	5	0.18	-80

4.3 Neurodynamical modeling of MSN

The MSN model in our paper was mainly from Wolf *et al.* [5]. Its soma had NaF, NaP, KAf, KAs, KIR, KRP, Leak, BKKCa, SKKCa, CaL1.2, CaL1.3, CaN, CaQ, CaR, CaT ion channels. The KAs, KRP, CaN, and CaL1.2 channels were described as partially inactivating channels, which were calculated by Equation (S-3); other channels were calculated by Equation (S-2). The SKKCA and BKKCA channels were calcium-dependent potassium channels, which were calculated by Equations (S-4∼S-6). The compartment current of MSN soma can be calculated by Equations (S-7∼S-8). MSN soma received GABA inhibition from 1 PV and 1 CB interneurons in NAc regions, which can be calculated according to Equations (S-9∼S-10) and Table (S2). The H-H model of MSN soma was described as Equation (S-13).

$$\begin{aligned}C_m \frac{dV_{NAc,MSN,soma}}{dt} &+ (I_{NaF} + I_{NaP} + I_{KAf} + I_{KAs} + I_{KIR} + I_{KRP} + I_{Leak} + I_{SKKCa} + I_{BKKCa} \\ &+ I_{CaL1.2} + I_{CaL1.3} + I_{CaN} + I_{CaQ} + I_{CaR} + I_{CaT}) + 10 \times I_{Compartment,dendrite \to soma} \\ &+ (I_{GABAa,\ NAc,PV \to NAc,MSN,soma} + I_{GABAa,\ NAc,CB \to NAc,MSN,soma}) = I_{Stimuli,\ NAc,MSN,soma}\end{aligned} \tag{S-13}$$

The parameters of ion channel kinetic in MSN soma was shown as Table (S3) for non-Ca^{2+} channels and Table (S4) for Ca^{2+} channels. Detailed modeling description can be found in previous papers [2, 3, 5].

Table S3 The non-calcium ionic channel parameter settings for MSN soma in NAc region of VTA-NAc-mPFC neurodynamical modeling [2].

**Ion Channel Type**	**Maximum Conductance** S/cm^2	**Partial Deactivating** a	$V_{1/2,m}$ mV	$V_{1/2,h}$ mV	m_k	h_k	**Reversal Potential** mV
NaF	1.5	NaN	-23.9	-62.9	-11.8	10.7	50
NaP	4e-5	NaN	-52.6	-48.8	-4.6	10	50
KAf	0.225	NaN	-10	-75.6	-17.7	10	-90
KAs	$\bar{g}_{KAs,DA}$ [1]	0.996	-27	-33.5	-16	21.5	-90
KIR	1.4e-4	NaN	-82	NaN	13	NaN	-90
KRP	0.001	0.7	-13.5	-54.7	-11.8	18.6	-90
Leak	11.5e-6	NaN	NaN	NaN	NaN	NaN	-70
BKKCa	0.001	NaN	NaN	NaN	NaN	NaN	-90
SKKCa	0.145	NaN	NaN	NaN	NaN	NaN	-90

[1] $\bar{g}_{KAs,DA} = 0.0104 + 0.0052 \times DARatio$, affected by dopamine concentrations.

Table S4 The calcium ionic channel parameter settings for MSN soma in NAc region of VTA-NAc-mPFC neurodynamical modeling [2].

**Ion Channel Type**	**Maximum permeability** cm/S	**Partial Deactivating** a	$V_{1/2,m}$ mV	$V_{1/2,h}$ mV	m_k	h_k
CaL1.2	$\overline{P}_{CaL1.2,DA}$ [2]	0.17	-8.9	-13.4	-6.7	11.9
CaL1.3	4.25e-7	NaN	-33	-13.4	-6.7	11.9
CaN	1.0e-5	0.21	-8.7	-74.8	-7.4	6.5
CaQ	6.0e-6	NaN	-9.0	NaN	-6.6	NaN
CaR	2.6e-5	NaN	-10.3	-33.3	-6.6	17
CaT	4e-7	NaN	-51.73	-80	-6.53	6.7

[2] $\overline{P}_{CaL1.2,DA} = 6.7 \times 10^{-6} - 6.7 \times 10^{-6} \times 0.2 \times DARatio$, affected by dopamine concentrations.

Compared with the MSN soma, ten identical dendrites had all types of ion channels except KRP channel. The kinetics of Ca^{2+} channels in dendrites were the same as the soma, which can be calculated as Table (S4). Although most of the kinetics of the non-Ca^{2+} channels in dendrite were consistent with those in soma as Table (S3), several parameters needed to be adjusted to match the experimental results [5]: 1) The maximum conductance of NaF channel was adjusted to 0.0195; 2) The maximum conductance of NaP channel was adjusted to 1.38e-7; 3) The maximum conductance of KAf channel was adjusted to 0.021; 4) The maximum conductance of KAs channel was adjusted to $\overline{g}_{KAs,DA} = 9.51 \times 10^{-4} + 4.755 \times 10^{-4} \times \mathrm{DARatio}$, which was affected by dopamine concentrations. The compartment current of MSN dendrite can be calculated similarly according to Equation (S-7). The synaptic currents of MSN dendrite were EPSCs from mPFC Pyra neurons (including AMPA and NMDA), which can be calculated according to Table (S2). The H-H model of MSN dendrite was described as Equation (S-14).

$$\begin{aligned} C_m \frac{dV_{NAc,MSN,dendrite}}{dt} &+ (I_{NaF} + I_{NaP} + I_{KAf} + I_{KAs} + I_{KIR} + I_{Leak} + I_{SKKCa} + I_{BKKCa} \\ &+ I_{CaL1.2} + I_{CaL1.3} + I_{CaN} + I_{CaQ} + I_{CaR} + I_{CaT}) + I_{Compartment,soma\rightarrow dendrite} \\ &+ \sum(I_{AMPA,\ mPFC,Pyra,soma\rightarrow NAc,MSN,dendrite} + I_{NMDA,\ mPFC,Pyra,soma\rightarrow NAc,MSN,dendrite}) \\ &= I_{Stimuli,\ NAc,MSN,dendrite} \end{aligned} \tag{S-14}$$

4.4 Neurodynamical modeling of interneurons in the NAc region

The interneurons in the NAc region consisted of 1 PV interneuron and 1 CB interneuron [2, 3]. Either was simplified to a 'point neuron' structure containing only one soma. The computational model was mainly based on the work of Wang *et al.* [14]. Inputs of dopamine concentration did not alter the kinetic parameters of these neurons.

The H-H model for PV interneuron in the NAc region was shown as Equation (S-15).

$$\begin{aligned} C_m \frac{dV_{NAc,PV}}{dt} &+ (I_{Na} + I_K + I_{Leak}) + \sum(I_{AMPA,\ mPFC,Pyra,soma\rightarrow NAc,PV} \\ &+ I_{NMDA,\ mPFC,Pyra,soma\rightarrow NAc,PV}) + I_{GABAa,\ NAc,CB\rightarrow NAc,PV} = I_{Stimuli,\ NAc,PV} \end{aligned} \tag{S-15}$$

Where, the kinetics of Na^+ , K^+ , and Cl^- (Leak) channels were calculated as in Table (S5). The PV interneuron in NAc region was excited by 20 Pyra neurons from mPFC region, and inhibited by 1 CB interneuron from NAc region. The parameters of stimulus current and synaptic currents can be found in Table (S1 and S2).

The H-H model for CB interneuron in the NAc region was shown as Equation (S-16).

$$\begin{aligned} C_m \frac{dV_{NAc,CB}}{dt} &+ (I_{Na} + I_K + I_{Ca} + I_{KCa} + I_h + I_{Leak}) \\ &+ \sum(I_{AMPA,\ mPFC,Pyra,soma\rightarrow NAc,CB} + I_{NMDA,\ mPFC,Pyra,soma\rightarrow NAc,CB}) \\ &+ I_{GABAa,\ NAc,PV\rightarrow NAc,CB} = I_{Stimuli,\ NAc,CB} \end{aligned} \tag{S-16}$$

Where, the kinetics of Na^+ , K^+ , and Cl^- (Leak) channels were similar to those of PV interneuron. Specifically, there were some other ion channel types, such as the

Table S5 The ionic channel parameter settings for PV interneuron in NAc region of VTA-NAc-mPFC neurodynamical modeling [2].

Ion Channel Type	Model	Ion Channel Type	Model
Na	$I_{Na} = \overline{g}_{Na} m_\infty^3 h(V_m - E_{Na})$<br>$m_\infty = \alpha_m/(\alpha_m + \beta_m)$<br>$\alpha_m = -\frac{0.1(V_m+35)}{\exp(-0.1(V_m+35))-1}$<br>$\beta_m = 4\exp(-\frac{V_m+60}{18})$<br>$\frac{dh}{dt} = 5(\alpha_h(1-h) - \beta_h h)$<br>$\alpha_h = 0.07\exp(-\frac{V_m+58}{20})$<br>$\beta_h = \frac{1}{\exp(-0.1(V_m+28))+1}$<br>$\overline{g}_{Na} = 35 mS \cdot cm^{-2}$<br>$E_{Na} = 55 mV$	K	$I_K = \overline{g}_K n^4 (V_m - E_K)$<br>$\frac{dn}{dt} = \alpha_n(1-n) - \beta_n n$<br>$\alpha_n = -\frac{0.01(V_m+34)}{\exp(-0.1(V_m+34))-1}$<br>$\beta_n = 0.125\exp(-\frac{V_m+44}{80})$<br>$\overline{g}_K = 9 mS \cdot cm^{-2}$<br>$E_K = -90 mV$
Leak	$I_{Leak} = \overline{g}_{Leak}(V_m - E_{Leak})$<br>$\overline{g}_{Leak} = 0.1 mS \cdot cm^{-2}$<br>$E_{Leak} = -65 mV$		

Ca^{2+} channel Ca, the calcium-dependent K^+ channel KCa, and hyperpolarization-activated cation current I_h. The kinetics of ion channels can be calculated as shown in Table (S6). The CB interneuron in NAc region was excited by 20 Pyra neurons from mPFC region, and inhibited by 1 PV interneuron from NAc region. The parameters of stimulus current and synaptic currents can be found in Table (S1 and S2).

Table S6 The ionic channel parameter settings for CB interneuron in NAc region of VTA-NAc-mPFC neurodynamical modeling [2].

Ion Channel Type	Model	Ion Channel Type	Model
Na	Same as Table (S5)	Ca	$I_{Ca} = \overline{g}_{Ca} m_\infty^2 (V_m - E_{Ca})$<br>$m_\infty = \frac{1}{1+\exp(-(V_m+20)/9)}$<br>$\overline{g}_{Ca} = 1 mS \cdot cm^{-2}$<br>$E_{Ca} = 120 mV$
K	Same as Table (S5)<br>$E_K = -85 mV$	KCa	$I_{KCa} = \overline{g}_{KCa} \frac{[Ca^{2+}]}{[Ca^{2+}]+K_D}(V_m - E_K)$<br>$\frac{d[Ca^{2+}]}{dt} = -\alpha_{Ca} I_{Ca} - \frac{[Ca^{2+}]}{\tau_{Ca}}$<br>$\alpha_{Ca} = 0.002$<br>$\tau_{Ca} = 80$<br>$K_D = 30$<br>$\overline{g}_{KCa} = 10 mS \cdot cm^{-2}$<br>$E_K = -85 mV$
Leak	Same as Table (S5)	h	$I_h = \overline{g}_h H(V_m - E_h)$<br>$\frac{dH}{dt} = \frac{H_\infty - H}{\tau_H}$<br>$H_\infty = \frac{1}{1+\exp((V_m+80)/10)}$<br>$\tau_H = \frac{200}{\exp((V_m+70)/20)+\exp(-(V_m+70)/20)} + 5$<br>$\overline{g}_h = 0.15 mS \cdot cm^{-2}$<br>$E_h = -40 mV$

4.5 Neurodynamical modeling of Pyra neurons in the mPFC region

There were 20 Pyra neurons in the mPFC region [2, 3]. The Pyra neuron had a structure of 'soma - proximal dendrite - distal dendrite'. The computational model was mainly based on the work of Wang *et al.* [14].

The H-H model for the soma of Pyra neuron in the mPFC region was shown as Equation (S-17).

$$
\begin{aligned}
&C_m \frac{dV_{mPFC,Pyra,soma}}{dt} + (I_{Na} + I_K + I_{Ca} + I_{Can} + I_{Leak}) + I_{Compartment,proximal \to soma} \\
&+ \sum I_{GABAa,\ mPFC,PV \to mPFC,Pyra,soma} + \sum I_{GABAa,\ mPFC,CB \to mPFC,Pyra,soma} \\
&= I_{Stimuli,\ mPFC,Pyra,soma}
\end{aligned} \tag{S-17}
$$

Where, the ion channel types included the Na^+ channel Na, the K^+ channel K, the high-threshold Ca^{2+} channel Ca, the slow calcium-dependent cationic channel Can, and leak channel Leak, which were calculated as shown in Table (S7). Its compartment current ($I_{Compartment,proximal \to soma}$) can be calculated according to Wang *et al* [14]. The soma of Pyra neuron was inhibited by their connected interneurons from mPFC regions (3 PV interneurons, 2 CB interneurons), whose parameters can be found in Table (S2).

Table S7 The ionic channel parameter settings for pyramidal neuron (soma) in mPFC region of VTA-NAc-mPFC model [2].

Ion Channel Type	Model	Ion Channel Type	Model
Na	$I_{Na} = \overline{g}_{Na} m_\infty^3 h(V_m - E_{Na})$<br>$m_\infty = \alpha_m/(\alpha_m + \beta_m)$<br>$\alpha_m = -\frac{0.1(V_m+31)}{\exp(-0.1(V_m+31))-1}$<br>$\beta_m = 4\exp(-\frac{V_m+56}{18})$<br>$\frac{dh}{dt} = 4(\alpha_h(1-h) - \beta_h h)$<br>$\alpha_h = 0.07\exp(-\frac{V_m+47}{20})$<br>$\beta_h = \frac{1}{\exp(-0.1(V_m+17))+1}$<br>$\overline{g}_{Na} = 55mS \cdot cm^{-2}$<br>$E_{Na} = 55mV$	K	$I_K = \overline{g}_K n^4(V_m - E_K)$<br>$\frac{dn}{dt} = 4(\alpha_n(1-n) - \beta_n n)$<br>$\alpha_n = -\frac{0.01(V_m+34)}{\exp(-0.1(V_m+34))-1}$<br>$\beta_n = 0.125\exp(-\frac{V_m+44}{80})$<br>$\overline{g}_K = 15mS \cdot cm^{-2}$<br>$E_K = -80mV$
Ca	$I_{Ca} = \overline{g}_{Ca} m_\infty^2 (V_m - E_{Ca})$<br>$m_\infty = \frac{1}{1+\exp(-(V_m+20)/9)}$<br>$E_{Ca} = 120mV$<br>$\overline{g}_{Ca} = 1.5 - 0.3 \times DARatio$<br>$\overline{g}_{Ca}$ affected by dopamine concentration	Can	$I_{Can} = \overline{g}_{Can} m^2 (V_m - E_{Can})$<br>$\frac{dm}{dt} = \frac{m_\infty - m}{\tau_{Can}}$<br>$m_\infty = \frac{\alpha[Ca^{2+}]}{\alpha[Ca^{2+}]+\beta}$<br>$\frac{d[Ca^{2+}]}{dt} = -\alpha_{Ca} I_{Ca} - \frac{[Ca^{2+}]}{\tau_{Ca}}$<br>$\alpha_{Ca} = 0.000667$<br>$\tau_{Can} = \frac{1}{\alpha[Ca^{2+}]+\beta}$<br>$\tau_{Ca} = 240$<br>$\alpha = 0.0056$<br>$\beta = 0.002$<br>$E_{Can} = -20mV$<br>$\overline{g}_{Can} = 0.025 - 0.005 \times DARatio$<br>$\overline{g}_{Can}$ affected by dopamine concentration
Leak	$I_{Leak} = \overline{g}_{Leak}(V_m - E_{Leak})$<br>$\overline{g}_{Leak} = 0.05mS \cdot cm^{-2}$<br>$E_{Leak} = -80mV$		

The H-H model for the proximal dendrite of Pyra neuron in the mPFC region was shown as Equation (S-18).

$$
\begin{aligned}
&C_m \frac{dV_{mPFC,Pyra,proximal}}{dt} + (I_{NaP} + I_{KS} + I_{Leak}) + I_{Compartment,soma \to proximal} \\
&+ I_{Compartment,distal \to proximal} = I_{Stimuli,\ mPFC,Pyra,proximal}
\end{aligned} \tag{S-18}
$$

Where, the ion channel types included the Na^+ channel NaP, the K^+ channel KS, and leak channel Leak, which were calculated as shown in Table (S8). Its compartment current came from its connected soma and distal dendrite, which can be calculated according to Wang *et al* [14]. The proximal dendrite of Pyra neuron did not connect to any synapse in our model.

Table S8 The ionic channel parameter settings for pyramidal neuron (proximal dendrite) in mPFC region of VTA-NAc-mPFC model [2].

Ion Channel Type	Model	Ion Channel Type	Model
NaP	$I_{NaP} = \bar{g}_{NaP} m_\infty^3 h(V_m - E_{Na})$<br>$m_\infty = \frac{1}{1+\exp(-(V_m+55.7)/7.7)}$<br>$\frac{dh}{dt} = \alpha_h(1-h) - \beta_h h$<br>$\alpha_h = 0.001\exp(-\frac{V_m+85}{30})$<br>$\beta_h = \frac{0.0034}{\exp(-(17+V_m)/10)+1}$<br>$\bar{g}_{NaP} = 0.15\mathrm{mS}\cdot\mathrm{cm}^{-2}$<br>$E_{Na} = 55\mathrm{mV}$	KS	$I_{KS} = \bar{g}_{KS} qr(V_m - E_K)$<br>$\frac{dq}{dt} = \frac{q_\infty - q}{\tau_q}$<br>$\frac{dr}{dt} = \frac{r_\infty - r}{\tau_r}$<br>$q_\infty = \frac{1}{1+\exp(-(V_m+34)/6.5)}$<br>$\tau_q = \frac{8}{\exp(-((V_m+55)/30))+\exp((V_m+55)/30)}$<br>$r_\infty = \frac{1}{1+\exp((V_m+65)/6.6)}$<br>$\tau_r = \frac{100}{1+\exp(-((V_m+65)/6.8))} + 100$<br>$E_K = -80\mathrm{mV}$<br>$\bar{g}_{KS} = 16 - 8\times \mathrm{DARatio}$<br>$\bar{g}_{KS}$ affected by dopamine concentration
Leak	Same as Table (S7)		

The H-H model for the distal dendrite of Pyra neuron in the mPFC region was shown as Equation (S-19).

$$
\begin{aligned}
C_m & \frac{dV_{mPFC,Pyra,distal}}{dt} + (I_A + I_{Ca} + I_{Leak}) + I_{Compartment,proximal\rightarrow distal} \\
& + \sum (I_{AMPA,\ mPFC,Pyra,soma\rightarrow mPFC,Pyra,distal} + I_{NMDA,\ mPFC,Pyra,soma\rightarrow mPFC,Pyra,distal}) \\
& = I_{Stimuli,\ mPFC,Pyra,distal}
\end{aligned} \tag{S-19}
$$

Where, the ion channel kinetics were shown in Table (S9). Its compartment current came from its connected proximal dendrite, which can be calculated according to Wang *et al* [14]. The distal dendrite of Pyra neuron was excited by their connected Pyra neurons from mPFC regions (other 19 Pyra neurons, did not consider self-connection), whose parameters can be found in Table (S2).

4.6 Neurodynamical modeling of interneurons in the mPFC region

The interneurons in the mPFC region consisted of 3 PV interneuron and 2 CB interneuron [2, 3]. The H-H model of PV interneuron in mPFC was shown as Equation (S-20), whose ion channel kinetics were same as those of PV interneuron in NAc region (See Table S5). Either of PV interneurons in mPFC was excited by the 20 Pyra neurons from mPFC region, and inhibited by the other 2 PV interneurons and 2 CB interneurons from mPFC region, whose parameters can be found in Table (S2).

$$
\begin{aligned}
C_m & \frac{dV_{mPFC,PV}}{dt} + (I_{Na} + I_K + I_{Leak}) + \sum (I_{AMPA,\ mPFC,Pyra,soma\rightarrow mPFC,PV} \\
& + I_{NMDA,\ mPFC,Pyra,soma\rightarrow mPFC,PV}) + \sum I_{GABAa,\ mPFC,PV\rightarrow mPFC,PV} \\
& + \sum I_{GABAa,\ mPFC,CB\rightarrow mPFC,PV} = I_{Stimuli,\ NAc,PV}
\end{aligned} \tag{S-20}
$$

Table S9 The ionic channel parameter settings for pyramidal neuron (distal dendrite) in mPFC region of VTA-NAc-mPFC model [2].

Ion Channel Type	Model	Ion Channel Type	Model
A Type K^+	$I_A = \bar{g}_A a^4 b(V_m - E_K)$<br>$\frac{da}{dt} = \frac{a_\infty - a}{\tau_a}$<br>$\frac{db}{dt} = \frac{b_\infty - b}{\tau_b}$<br>$a_\infty = \frac{1}{1+\exp(-(V_m+60)/8.5)}$<br>$\tau_a = 0.37 + \frac{1}{\exp((V_m+35.8)/19.7)+\exp(-(V_m+79.7)/12.7)}$<br>$b_\infty = \frac{1}{1+\exp((V_m+78)/6)}$<br>$\tau_b = 19 + \frac{1}{\exp((V_m+46)/5)+\exp(-(V_m+238)/37.5)}$<br>$\bar{g}_A = 1\mathrm{mS}\cdot\mathrm{cm}^{-2}$<br>$E_{Na} = -80\mathrm{mV}$	Leak	Same as Table (S7)
Ca	$I_{Ca} = \bar{g}_{Ca} m_\infty^2 (V_m - E_{Ca})$<br>$m_\infty = \frac{1}{1+\exp(-(V_m+20)/9)}$<br>$E_{Ca} = 120\mathrm{mV}$<br>$\bar{g}_{Ca} = 0.25 - 0.05 \times \mathrm{DARatio}$<br>$\bar{g}_{Ca}$ affected by dopamine concentration		

Similarly, The H-H model of CB interneuron in mPFC was shown as Equation (S-21), whose ion channel kinetics were same as those of CB interneuron in NAc region (See Table S6). Either of CB interneurons in mPFC was excited by the 20 Pyra neurons from mPFC region, and inhibited by 3 PV interneurons and the other CB interneuron from mPFC region, whose parameters can be found in Table (S2).

$$\begin{aligned} C_m \frac{dV_{mPFC,CB}}{dt} &+ (I_{Na} + I_K + I_{Ca} + I_{KCa} + I_h + I_{Leak}) \\ &+ \sum (I_{AMPA,\ mPFC,Pyra,soma\to mPFC,CB} + I_{NMDA,\ mPFC,Pyra,soma\to mPFC,CB}) \\ &+ \sum I_{GABAa,\ mPFC,PV\to mPFC,CB} + I_{GABAa,\ mPFC,CB\to mPFC,CB} = I_{Stimuli,\ mPFC,CB} \end{aligned} \tag{S-21}$$

4.7 Morphological characteristics

According to the Equation (S-1), the H–H model calculated the currents per unit membrane area as $\mu A\cdot(cm)^{-2}$. Since the four neuronal types (MSN, PV interneurons, CB interneurons, and pyramidal neurons) in our model had different morphological characteristics with different neuronal membrane areas, we had to define the total membrane areas before calculating the whole-cell currents.

Based on previous literatures [5, 12], we considered the soma and dendrites as the cylinder shapes whose ion channels and other currents were only on the lateral side. Then, their membrane area can be calculated as $S = \pi ld$, where 'd' was the diameter of the base of the cylinder, and 'l' was the height. These parameters were measured in neurons in vitro [5, 12]. Given the fact that we simplified the structures, we adjusted some parameters from physiological experiments in order to ensure that the simplified modeling membrane area was approximately the same as the experimentally measured values. The length and diameter we used in this paper were shown in Table (S10).

4.8 Experimental replication for robustness

There are three stochastic variables in our model: 1) connection strength matrix; 2) stimulus current; and 3) dopamine concentration. In order to get robust conclusions, especially to avoid the influence of the three stochastic variables, repeated experiments of 9 replicates for the trial were conducted based on Table (S11). The simulated

Table S10 The morphological characteristics of different neuronal types for the VTA-NAc-mPFC neurodynamical modeling [3].

Neuronal Type	Location	Length l (μm)	Diameter d (μm)	Surface Area (cm^2)	Reference
MSN	Soma	20	20	1.257e-5	[5]
MSN	Dendrite	395.2	0.72	8.939e-6	[5]
PV Interneuron	-	35	35	3.848e-5	[12]
Interneuron	-	35	35	3.848e-5	[12]
Pyra	Soma	25	25	1.963e-5	[12]
Pyra	Proximal Dendrite	400	2.6	3.267e-5	[12]
Pyra	Distal Dendrite	400	2.6	3.267e-5	[12]

results were statistically analyzed to ensure the significances of the findings. Here, the dopamine concentrations were set up to 4 groups (See **Figure 1d** in the manuscript): Low (randomly varying from 0 to 0.25), Medium (randomly varying from 0.25 to 0.50), High (randomly varying from 0.50 to 0.75), Full (randomly varying from 0.75 to 1.00), following a uniform distribution. The rodent tended to exhibit more intensely depressive-like behaviors and electrophysiological patterns when the dopamine concentration was lower. The simulation method was using the random seed function 'rng' in MATLAB, which ensured the reproducible results. For example, although the stimuli current varied dynamically over the range, when the random seed of the stimulus current was fixed, the dynamics of the stimuli current over time for 9 replicates of the same neuron exhibited exactly the same pattern, which was equivalent to fixing the stimulus current unchanged within the same trial.

Table S11 The parameter settings for repeatability experiment of VTA-NAc-mPFC model [2].

Dopamine Concentration	Fixed Variable	Changing Variable	Replicates
DARatio $\sim$ U$[0, 0.25]$, 'Low' concentration<br>DARatio $\sim$ U$[0.25, 0.50]$, 'Medium' concentration<br>DARatio $\sim$ U$[0.50, 0.75]$, 'High' concentration<br>DARatio $\sim$ U$[0.75, 1.00]$, 'Full' concentration	Stimuli Currents	Dopamine Concentration<br>Connection Strength	n = 9

4.9 Initial values

The initial membrane potentials for MSN soma and dendrites in the NAc region were set to -90 mV. The initial membrane potential for Pyra soma in mPFC region was -64.8 mV, with -64 mV for its proximal dendrite and distal dendrite. The initial membrane potentials for all the interneurons were set up to -64 mV. The initial values of the gate variables in Equations (S-2, S-10) were set up to 0, meaning that the status of these gates were all fully closed at the initial moment. The numerical solution of our model was using 2nd- or 3rd-Order Runge-Kutta method in Matlab ('ode23' function), with a step size of 0.02, which was equivalent to setting the sampling frequency to 50,000 Hz.

5 Neural energy model

In order to investigate the energy coding patterns of our VTA-NAc-mPFC neural microcircuit model (Equation S-1), we augmented the Moujahid's neural energy model [3, 15]. In Moujahid's original neural energy model, the neural power $\mathrm{H_{All}}$ of the basic

H-H model ($C_m \frac{dV_m}{dt} + \sum I_{Ions} = I_{Stimuli}$, only including K^+ channel, Na^+ channel, Leak channel, and stimulus current) can be calculated by Equation (S-22). However, in our model, we have multiple types of currents, i.e., Ca^{2+} currents defined by GHK models, compartment currents, and synaptic currents. Thus, we had augmented the Moujahid's neural energy model [3]. The detailed description can be found in [3, 15].

$$H_{All} = H_{Stimuli} - \sum H_{Ions} = V_m I_{Stimuli} - \sum I_{Ions}(V_m - E_{Ions}) \tag{S-22}$$

Regarding the Ca^{2+} currents defined by GHK models, although the calculation was based on the permeability (Equations S-4∼S-6), their reversal potentials can be calculated by Equation (S-23) [4].

$$E_{ions,\ GHK} = \frac{G_{ions,\ GHK} V_m - I_{ions,\ GHK}}{G_{ions,\ GHK}} \tag{S-23}$$

Where, $G_{ions,\ GHK}$ denoted the dynamically changing conductance of the ion channel currents, and it can be calculated by Equation (S-24). All the parameters in Equation (S-24) can be found in Equations (S-4∼S-6).

$$\begin{aligned} G_{ions,\ GHK} = {} & P_{ions,\ GHK} z^2_{ions,\ GHK} \frac{F^2}{RT} \frac{[ions]_{in} - [ions]_{out} \cdot \exp(-z_{ions,\ GHK} F V_m / RT)}{1 - \exp(-z_{ions,\ GHK} F V_m / RT)} \\ & - P_{ions,\ GHK} z^3_{ions,\ GHK} \frac{F^3}{R^2 T^2} ([ions]_{in} - [ions]_{out}) \frac{V_m \exp(-z_{ions,\ GHK} F V_m / RT)}{1 - \exp(-z_{ions,\ GHK} F V_m / RT)} \end{aligned} \tag{S-24}$$

Then, the neural power of the Ca^{2+} currents defined by GHK models can be calculated by Equation (S-25).

$$\begin{aligned} H_{ions,\ GHK} &= I_{ions,\ GHK}(V_m - E_{ions,\ GHK}) = I_{ions,\ GHK}\left(V_m - \frac{G_{ions,\ GHK} V_m - I_{ions,\ GHK}}{G_{ions,\ GHK}}\right) \\ &= \frac{I^2_{ions,\ GHK}}{G_{ions,\ GHK}} \end{aligned} \tag{S-25}$$

Regarding the synaptic currents, only its effect on the H-H circuit (i.e., electrical power) were considered in our paper, without taking into account protein anabolic consumption during synaptic transmission. In this way, the synaptic currents were equivalent to an externally input current (See **Fig. 1e**), and they can be calculated as Equation (S-26).

$$H_{Synapse} = \sum I_{Synapse} V_m = (\sum I_{AMPA} + \sum I_{NMDA} + \sum I_{GABA}) V_m \tag{S-26}$$

Regarding the compartment currents, similarly, they can be considered as externally input current according to the H-H circuit structure (See **Fig. 1e**), even though they were induced by the membrane potential gradients from neighboring structures. They can be calculated as Equation (S-27).

$$H_{Compartment} = V_m I_{Compartment} \tag{S-27}$$

Thus, the neural power of our VTA-NAc-mPFC neural microcircuit can be described as Equation (S-28).

$$H_{All} = H_{Stimuli} + \sum H_{Compartment} + \sum H_{Synapses} - \sum H_{Ions} \tag{S-28}$$

In the current paper, we studied the neural energy coding patterns for the VTA-NAc-mPFC neural microcircuit defined as $\mathrm{W_{All}} = \int_0^{3,000\mathrm{ms}} \mathrm{H_{All}(t)dt}$, i.e., measuring the total energy consumption over 3,000 ms that the neurons required. In previous work [3], we mainly studied the ion channel energy consumption patterns defined as $\mathrm{W_{Ion}} = \int_0^{3,000\mathrm{ms}} \mathrm{H_{Ion}(t)dt}$, i.e., measuring how much neural energy the ion channels theoretically required to keep the neuronal activities.

Code availability

The MATLAB codes that support the findings of this study are available at GITHUB (https://github.com/Yuanxi-Li/MDD-NeuralEnergy).

References

[1] Russo, S. J. & Nestler, E. J. The brain reward circuitry in mood disorders. *Nature reviews neuroscience* **14**, 609–625 (2013).

[2] Li, Y. *et al.* Dopamine-mediated major depressive disorder in the neural circuit of ventral tegmental area-nucleus accumbens-medial prefrontal cortex: from biological evidence to computational models. *Frontiers in Cellular Neuroscience* **16**, 923039 (2022).

[3] Li, Y., Zhang, B., Liu, Z. *et al.* Neural energy computations based on hodgkin-huxley models bridge abnormal neuronal activities and energy consumption patterns of major depressive disorder. *Computers in Biology and Medicine* **166**, 107500 (2023).

[4] De Schutter, E. & Smolen, P. Calcium dynamics in large neuronal models. *Methods in neuronal modeling: From ions to networks* **2** (1998).

[5] Wolf, J. A. *et al.* Nmda/ampa ratio impacts state transitions and entrainment to oscillations in a computational model of the nucleus accumbens medium spiny projection neuron. *Journal of Neuroscience* **25**, 9080–9095 (2005).

[6] Heinemann, S. H., Terlau, H., Stühmer, W. *et al.* Calcium channel characteristics conferred on the sodium channel by single mutations. *Nature* **356**, 441–443 (1992).

[7] Rall, W. Theory of physiological properties of dendrites. *Annals of the New York Academy of Sciences* **96**, 1071–1092 (1962).

[8] Rall, W. Electrophysiology of a dendritic neuron model. *Biophysical journal* **2**, 145 (1962).

[9] Rall, W. Branching dendritic trees and motoneuron membrane resistivity. *Experimental neurology* **1**, 491–527 (1959).

[10] Ermentrout, B. & Terman, D. H. *Mathematical foundations of neuroscience* Vol. 35 (Springer, New York, 2010).

[11] Destexhe, A., Mainen, Z. F., Sejnowski, T. J. *et al.* Kinetic models of synaptic transmission. *Methods in neuronal modeling* **2**, 1–25 (1998).

[12] Durstewitz, D., Seamans, J. K. & Sejnowski, T. J. Dopamine-mediated stabilization of delay-period activity in a network model of prefrontal cortex. *Journal of neurophysiology* (2000).

[13] Konstantoudaki, X., Papoutsi, A., Chalkiadaki, K. *et al.* Modulatory effects of inhibition on persistent activity in a cortical microcircuit model. *Frontiers in neural circuits* **8**, 7 (2014).

[14] Wang, X.-J., Tegnér, J., Constantinidis, C. *et al.* Division of labor among distinct subtypes of inhibitory neurons in a cortical microcircuit of working memory. *Proceedings of the National Academy of Sciences* **101**, 1368–1373 (2004).

[15] Moujahid, A., d'Anjou, A., Torrealdea, F. *et al.* Energy and information in hodgkin-huxley neurons. *Physical Review E* **83**, 031912 (2011).
