# Supplementary material for: Neural energy coding patterns of dopaminergic neural microcircuit and its impairment in major depressive disorder: A computational study
Source: PLoS Comput Biol. 2025 Apr 7;21(4):e1012961. doi: 10.1371/journal.pcbi.1012961 (PMC12002636; doi:10.1371/journal.pcbi.1012961)
Supplement: S1 Fig — Since the dopamine concentration was set as a uniform distribution within a range, the midpoint of the interval was taken as the dopamine concentration for plotting (Low – 0.125, Medium – 0.375, High – 0.625, Full – 0.875). p values are not corrected here. (PDF) [file pcbi.1012961.s002.pdf]

Neural energy coding patterns of dopaminergic neural microcircuit and its impairment in major depressive disorder: A computational study

Supplementary Figure

Yuanxi Li[1, 5, *], Bing Zhang[2, 3, 5], Jinqi Liu[4], Rubin Wang[1, *]

1. Institute for Cognitive Neurodynamics, School of Mathematics, East China University of Science and Technology, Shanghai, China
2. Department of Anesthesiology, Obstetrics and Gynecology Hospital of Fudan University, Fudan University, Shanghai, China
3. Shanghai Key Laboratory of Maternal Fetal Medicine, Shanghai Institute of Maternal-Fetal Medicine and Gynecologic Oncology, Department of Anesthesiology, Clinical and Translational Research Center, Shanghai First Maternity and Infant Hospital, Tongji University School of Medicine, Shanghai, China
4. University of Rochester, Rochester, New York, USA
5. These authors contributed equally to the manuscript

* Corresponding author:
dr.yuanxili@gmail.com (Y.L.),
rbwang@ecust.edu.cn (R.W.).

Contributing authors:
bingozzz@126.com (B.Z.),
jliu161@u.rochester.edu (J.L.).

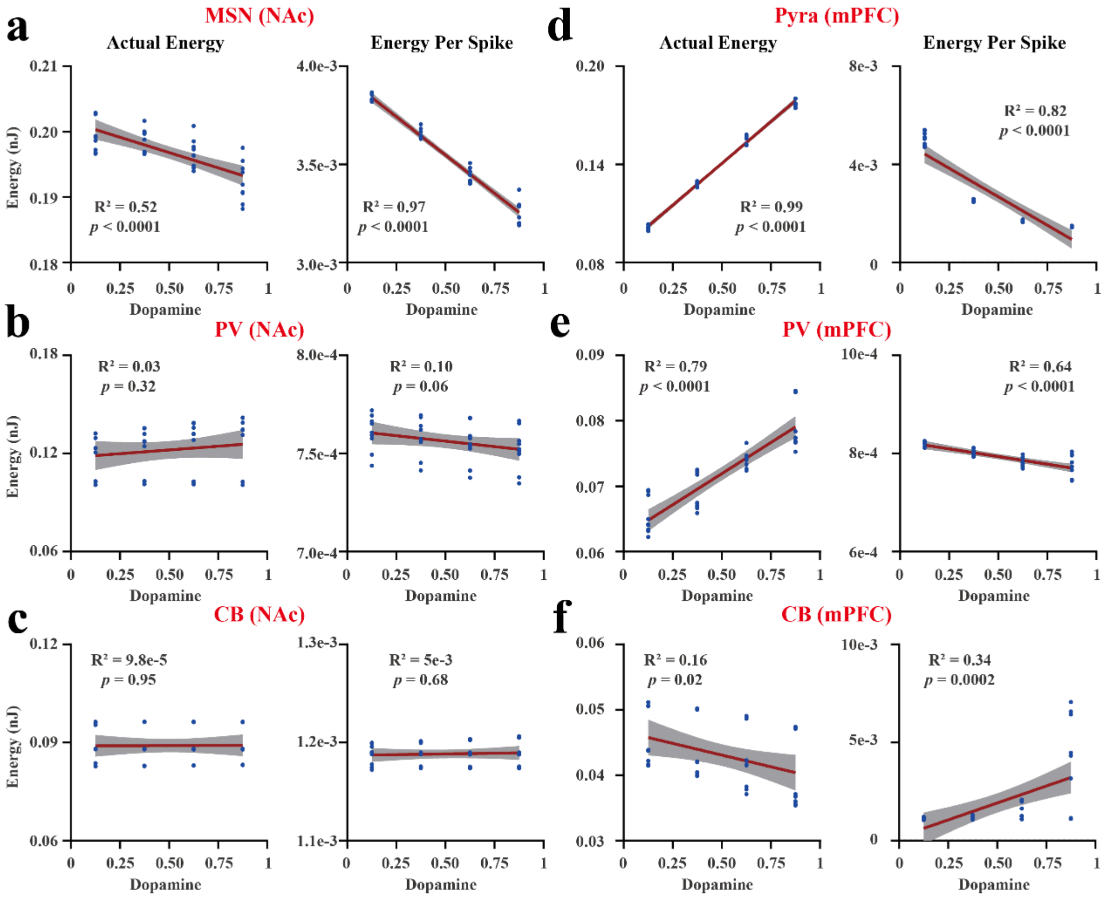

Fig S1 The regression lines between dopamine concentration and actual energy or energy per spike in different neuronal types. Since the dopamine concentration was set as a uniform distribution within a range, the midpoint of the interval was taken as the dopamine concentration for plotting (Low – 0.125, Medium – 0.375, High – 0.625, Full – 0.875). *p* values are not corrected here.
