# Supplementary material for: Neural energy coding patterns of dopaminergic neural microcircuit and its impairment in major depressive disorder: A computational study
Source: PLoS Comput Biol. 2025 Apr 7;21(4):e1012961. doi: 10.1371/journal.pcbi.1012961 (PMC12002636; doi:10.1371/journal.pcbi.1012961)
Supplement: Attachment — Submitted filename: Response Letter.pdf [file pcbi.1012961.s005.pdf]

Point-by-point Response Letter

Dear Editors and Reviewers,

We appreciate your thoughtful consideration of our manuscript and the constructive comments from the reviewer. Below are our point-by-point responses (in black) to the comments (in light blue) from the reviewer. The revisions in our updated manuscript are highlighted in blue (Please go to the end of this .pdf file to view the marked manuscript).

Reviewer #1

The paper entitled "Neural energy coding patterns of dopaminergic neural microcircuit and its impairment in major depressive disorder: A computational study" by Li et al is an advance on prior work from this group which developed neural network modeling of the VTA-NAc-mPFC neural microcircuit using the basic Hodgkin-Huxley (H-H) model but allowing it to be linked to neurotransmitter level, which in their case is dopamine. This advancement thereby allows neurotransmitter level to be linked to the H-H estimates which are largely based on ionic currents. The current work is an extension of the prior model to calculate neural energy based on the VTA-NAc-mPFC neural microcircuit model, specifically instantaneous neural power corresponding, respectively, to Equations (1) and (2) in their paper. They studied the neural energy consumption under different dopamine inputs and related their neurotransmitter levels to energy consumed and energy/spike. In the current work, the 'Low' dopamine was considered as the MDD group, whereas the 'Full' dopamine was considered as the NC group. Quite sadly, no specific conclusion was made about MDD vs. NC per se. Instead, very general conclusions were made. While there are no major errors in the work, as the authors themselves state, the work is purely computational and thus it is difficult to assess how their work could be useful in the knowledge of neurotransmitter levels vs. neuroenergetics in health and disease. Please note that since both of these metrics (neurotransmitter vs. neuroenergetics) can be measured in vivo, it is important to reveal how their work is relevant in MDD, but also other psychiatric disorders.

Our reply: We thank the reviewer for the comment. We partly agree with the reviewer's comment that 'Please note that since both of these metrics (neurotransmitter vs. neuroenergetics) can be measured in vivo, it is important to reveal how their work is relevant in MDD, but also other psychiatric disorders.'. Although some probes, such as those developed by Yulong Li's lab for dopamine and serotonin (5-HT), enable *in vivo* measurement of neurotransmitter levels, and large-scale BOLD-fMRI patterns in major depressive disorder (MDD) have been reported in some literatures, as far as we know, no existing experimental technique can simultaneously capture the dynamics between membrane potentials, neurotransmitter levels, ion currents kinetics, and synaptic currents kinetics within a specific neural circuit. Therefore, given the limitations of current techniques, it may be impossible to fully achieve the reviewer's request to 'assess how their work could be useful in the knowledge of neurotransmitter levels vs. neuroenergetics in health and disease'. This limitation also underlies our attemption to use theoretical models to investigate energy consumption patterns of the VTA-NAc-mPFC neural circuit.

However, although our current model is theoretical, it was developed based on existing experimental data and biological processes, successfully replicating the dynamic characteristics of the VTA-NAc-mPFC neural circuit, including membrane potential, ion channel kinetics, synaptic dynamics, and field potentials [1]. Notably, our model is the first to theoretically explore neural energy consumption characteristics in MDD at the neural circuit level. As a preliminary study in this field, we acknowledge these limitations of our models (as discussed in the last paragraph of **Discussion** section), and plan to design further experiments in the future to validate our findings.

Comment 1: While it is stated that Equations (1) and (2) respectively reflect the prior and current work, it is important to show how ionic currents in the H-H equations are related to levels of neurotransmitter. Some clear statements in the Introduction and Discussion, with supporting supplementary information, about the relation between ionic currents vs. membrane potential (which is very well known from prior work on glutamatergic and GABAergic systems) and neurotransmitter level vs. membrane potential (which is less well known and is the novelty of their work). It is crucial to identify these relations that are widely applicable across all neurotransmitter systems.

Our reply: We thank the reviewer for this valuable suggestion. In our previous study [1], we developed a mathematical model of the VTA-NAc-mPFC neurocircuit and systematically analyzed its neurodynamics, including the regulatory relationships among dopamine concentration inputs, ion channel kinetics (and currents), neurotransmitter-receptor binding kinetics (and synaptic currents), membrane potentials and local field potentials.

For instance, we examined the dynamics of KAs currents (a slow A-type sodium channel) in medium spiny neurons under varing dopamine concentration, and found that as dopamine levels increased, the amplitude of this current also increased. Regarding synaptic currents, we computed the correlation coefficient between membrane potential and synaptic currents. We then observed that the correlations between EPSCs and membrane potentials in pyramidal neurons were significantly positively correlated with dopamine concentration input, suggesting a regulatory role of glutamate neurotransmitter in the VTA-NAc-mPFC neural microcircuit.

These calculations were performed in a dynamical neural system (where the membrane potentials and currents change continuously over time), rather than in a static system (where membrane potentials or currents are fixed to specific levels, as is common in electrophysiological experiments). All modeling details and observations are provided in our previous study [1] and in the **Supplementary File 1** of the current manuscript.

Since the primary focus of this manuscript is the theoretical calculation and exploration of neural energy consumption patterns, we initially assumed that readers would be familiar with our previous neurodynamic modeling. Therefore, we did not include detailed descriptions of those aspects in our original submission. In the revised manuscript, we have added relevant discussions of our previous findings in the **Introduction** and **Discussion** sections to provide additional context.

Comment 2: It is assumed that most readers know distributions of different neurotransmitters in VTA, NAc, mPFC, etc, or do the authors assume the ratio of different neurotransmitters to be the same across these regions? Both can be true. At present the authors don't speak about different neurotransmitter levels in any region. Please let the readers know in Methods using absolute units (mM) how major neurotransmitters (glutamate, GABA, dopamine, serotonin, etc) vary across these regions. This information should then be showed in Methods as varying model equations for each region, as one of their conclusions is that parvalbumin interneuron (PV) and calbindin interneuron (CB) in NAc have different behaviors. Why?

Our reply: The regional neurotransmitter concentrations of the glutamate, GABA and dopamine were not directly incorporated into our model [1], as our VTA-NAc-mPFC model was developed based on the Hodgkin-Huxley model [2] and the neurotransmitter-receptor binding kinetics models [3].

More specifically, our model considered two major groups of neurotransmitters: ionotropic receptors, including the AMPA receptor (AMPAR), NMDA receptor (NMDAR) and $GABA_a$ receptor ($GABA_aR$); and G protein-coupled receptors, including the dopamine receptor (D1 and D2). The effects of AMPAR, NMDAR and $GABA_aR$ were presented as synaptic currents (EPSCs and IPSCs), whose kinetics can be measured through eletrophysiological experiments, including parameters such as maximum conductances G_{max}, half-activated voltages $V_{1/2}$, time constants τ (or

alternatively, activation rates α and deactivation rates β) and the reversal membrane potentials E_{rev} . By incorporting these experimentally derived measurements, we were able to simulate synaptic currents without explicitly considering neurotransmitter concentrations [3]. However, to account for the diversity of neurotransmitter dynamics and synaptic currents across different brain regions and neuronal types, we referred to relevant literature [3, 4], and assigned distinct parameter values to different synaptic current types (see **Table S2** in **Supplementary File 1**).

Additionally, the effects of dopamine (and dopamine receptors) were modeled based on previous studies [4-8], where dopamine concentrations were not explicitly mentioned. In our model, parameters representing dopamine receptor effects were also region- and neuron-type-specific (see the section **Dopamine Input** in **Supplementary File 1**).

Regarding the reviewer's comment: 'This information should then be showed in Methods as varying model equations for each region, as one of their conclusions is that parvalbumin interneuron (PV) and calbindin interneuron (CB) in NAc have different behaviors. Why?' – these distinctions arose from differences in ion channel kinetics, synaptic current kinetics, and connectivity patterns between PV and CB interneurons in the NAc region (see **Table S2, S5, S6** and section **4.4** in **Supplementary File 1;** We built distinct neurodynamical models for the two neuronal types). Due to these intrinsic differences, as well as modulation by varying dopamine input within the neural network, PV and CB interneurons exhibited distinct firing patterns and energy consumption characteristics. We have added the interpretation in Discussion section (paragraph 3) in the revised manuscript.

Comment 3: The authors state that "Numerous experiments have found that the behavioral characteristics of major depressive disorder (MDD) animals are usually associated with abnormal neural activity patterns and brain energy metabolism." Please clarify by which methods these energetic values were measured, or are they just referring to computational predictions? Unclear.

Our reply: In section **Introduction** (Paragraph 2), we summarized experimental evidence demonstrating abnormal energy metabolism patterns in MDD across molecular, cellular, and large-scale neural network levels in both animals and humans:

'*……, which has been observed in abundant studies: 1) At the molecular level, multi-omics results have exhibited differences in the levels of energy-metabolism-related metabolites in the cerebrospinal fluid of MDD animals and human compared to normal control (NC) group; 2) At the cellular level, the upregulated astroglial Kir4.1 channel leads to abnormal neuronal bursts in depression , while astrocytes are also responsible for supplying energy to neurons, suggesting a possible difference in their neural energy coding patterns; and 3) At the large-scale brain network level, functional magnetic resonance imaging (fMRI) experiments have demonstrated different blood oxygen level-dependent (BOLD) signals and functional connectivity among regions in MDD brains.*'

Additionally, we added the interpretation of our previous computational study [9] in **Introduction** section, where we identified some changes in energy consumption patterns (energy consumption by ion channels) based on our VTA-NAc-mPFC computational model. Thus, both computational and experimental evidence support our point.

Comment 4: The authors state that they "not only uncovered the neural energy coding patterns for the VTA-NAc-mPFC neural microcircuit, but also presented a novel pipeline for the study of MDD based on the neural coding theory and neural energy coding theory." The former is more important than the latter. So please compare for each region and the circuit together for clarifying this statement further. The Abstract should not have vague statements as nothing conclusive is stated about MDD although it's in the title.

Our reply: We thank the reviewer for this helpful suggestion. In the revised manuscript, we have added the interpretation in the **Discussion** section (paragraph 3). And we also interpreted our main findings for the two important neuronal types (MSN and pyramidal neurons) in more detailed in the **Abstract** section.

Comment 5: Although a recent discovery, it has been shown that dopaminergic neurons allow co-transmission of glutamate across phyla, from flies to humans. How does their conclusion change if certain fraction of dopaminergic neurons inherently co-transmit glutamate? This should at least be a discussion point of weaknesses.

Our reply: Since our VTA-NAc-mPFC neural microcircuit model is a simplified representation, we focused only on the major factors, including key neuronal types, ion channels, and neurotransmitters. While we do not currently have expertise in the specific field mentioned by the reviewer, we acknowledge the importance of this aspect and will consider it in future studies. Additionally, we have noted this limitation in the section **Discussion** (last paragraph).

Comment 6: In MDD and other psychiatric studies, the attention seems to be on the unique cellular architecture of dopaminergic neurons, specifically their large and complex arborization of axons, which puts these neurons under a heavy energy budget limit and thus makes them particularly susceptible to factors that contribute to subsequent cell death. However, if these cells are only 1% or 5% across regions, for their loss to have energetic impact on that region depends on their energy budget. The field of energy budget for glutamatergic and GABAergic systems, across phyla and in the human brain, have been well developed. How do the energy budgets of these neuronal systems vary?

Our reply: We appreciate the reviewer's concern. In our current study, we observed similar results. For example, the energy consumption of the medium spiny neuron (MSN) (**Fig.R1a**, also shown in **Fig. 3b** of the manuscript) was higher than that of PV interneurons in the mPFC region (**Fig.R1b**, also shown in **Fig. 5e** in the manuscript), likely due to the complex structures of MSN (**Fig. 1b**).

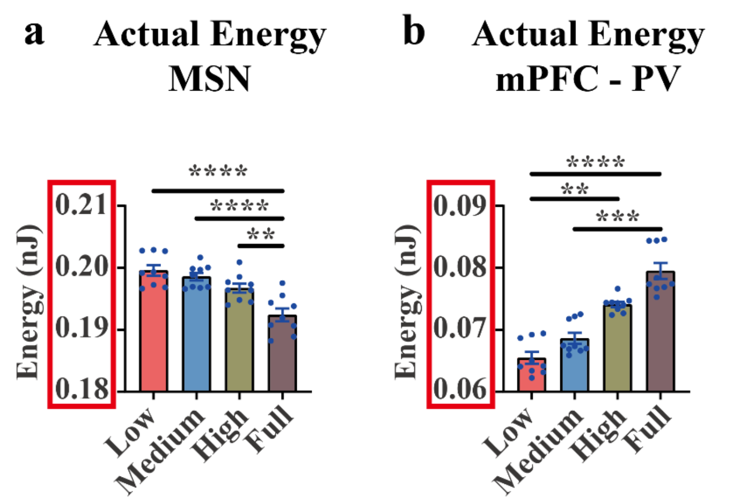

Figure R1 The actual neural energy results of the whole MSN (**a**) and the PV interneuron in the mPFC region (**b**), which are from the Fig. 3b and Fig. 5e.

Regarding the reviewer's comment 'However, if these cells are only 1% or 5% across regions, for their loss to have energetic impact on that region depends on their energy budget.', we agree with this point. However, addressing this question is beyond the scope of our current study, as our VTA-NAc-mPFC model represents a simplified neural microcircuit rather than a large-scale network model (i.e., one that includes entire brain regions with realistic neuronal proportions). Nonetheless, we appreciate the reviewer for raising this important point, and we will consider addressing it in future research. We have noted this limitation in the section **Discussion** (last paragraph).

Comment 7: The section in Discussion that compares the energetics of different regions, it is necessary to convert the

nJ units to mircomole/g/min – which is the main unit for metabolic measures by in vivo methods. This is important to understand the impact of regional differences regardless of their neurotransmitter levels.

Our reply: We acknowledge the reviewer's concern regarding unit. However, our neural energy model was developed at the microcircuit level (VTA-NAc-mPFC neural microcircuit) rather than tissue-scale physiology, making direct conversion of nJ to mM/g/min fundamentally challenging without precise neuron density data per gram of tissue.

To address model validity, we employed an alternative cross-scale consistency check in our previous work [9]. Based on our current simulation results, under 'Full' dopamine condition, the total energy consumption of the 28-neuron VTA-NAc-mPFC network over 3,000 ms was calculated as 4.2 nJ (**Fig. 7i**). This corresponds to an average neuronal power of 0.05 nW/neuron.

Assuming murine and human neurons share comparable power demands (a conservative simplification), scaling this value to the human brain's ~86 billion neurons yields a total neuronal power of 4.3 W. Given that empirical estimates suggest human brain consumes ~20 W (with ~15 W attributed to gray matter), and considering that our model does not account for the energy consumption by protein synthesis, glial cell activities, and other metabolic processes, the energy result of 4.3W aligns with biological reality. Thus, our computational framework is in line with the emperical evidence.

Comment 8: Please avoid jargon before they are defined, for example, Pyra? I understand it's pyramidal neurons etc. So please have a table for all abbreviations used with units converted for comparisons.

Our reply: We appreciate the reviewer's attention to detail. The use of "Pyra" to abbreviate pyramidal neurons is an legacy problem from our prior work in this research series [1, 9]. To maintain the consistency within this series, we have kept this abbreviation. We have added an **Abbreviation** section in the revised manuscript to summarize all abbreviations.

Minor Comment 1: Fig 4 (glutamatergic) and Fig 5 (GABAergic) need to do comparisons of B and C figures for slopes and exponent etc

Our reply: <u>We have added the **Fig. S1** as a **Supplementary Figure** (we also attached **Fig. R2** here)</u>, illustrating the regression lines between dopamine concentration and both actual energy and energy per spike across different neuronal types. <u>We added the relevant interpretation it in the **Result** and **Discussion** sections.</u>

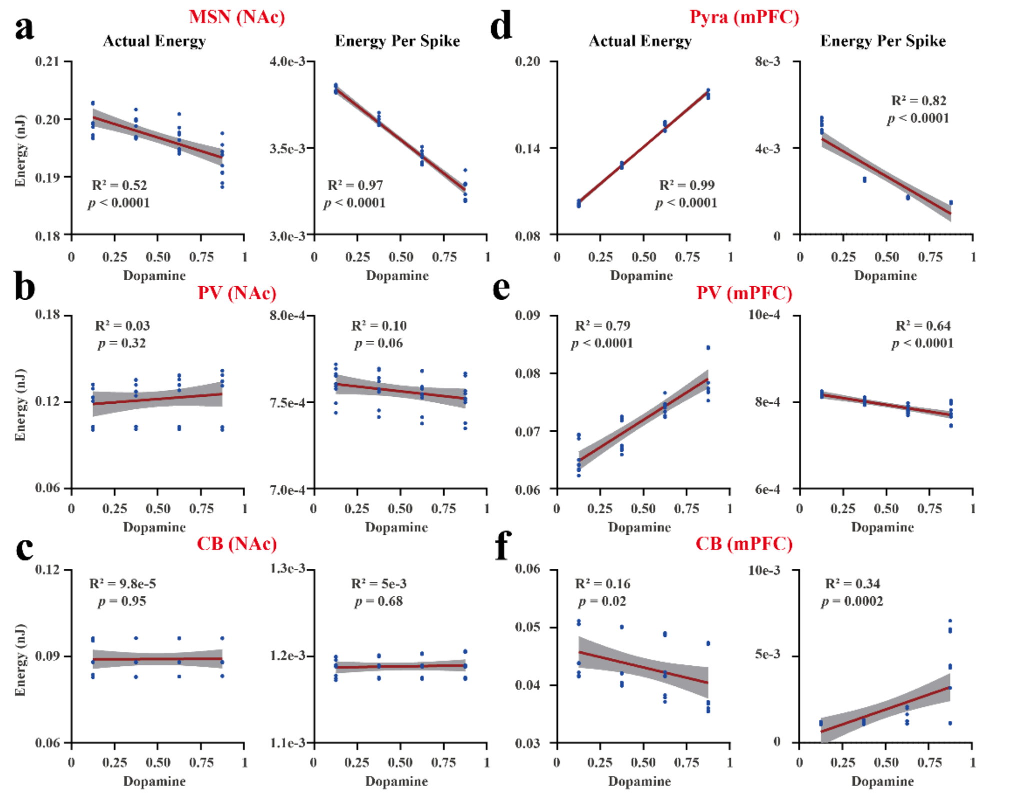

Figure R2 The regression lines between dopamine concentration and actual energy or energy per spike in different neuronal types. Since the dopamine concentration was set as a uniform distribution within a range, the midpoint of the interval was taken as the dopamine concentration for plotting (Low – 0.125, Medium – 0.375, High – 0.625, Full – 0.875). p values are not corrected here.

The results demonstrated that dopamine concentration selectively affected the energy consumption patterns of certain neuronal types, such as MSN (**a**), pyramidal neurons (**d**), PV interneuron (**e**) and CB interneuron (**f**) in the mPFC brain region. However, the energy consumption patterns of PV interneuron (**b**) and CB interneuron (**c**) in the NAc region was not significantly affected by dopamine, indicating a regional difference between the NAc and mPFC brain regions.

Minor Comment 2: Comparison of Fig 5/6 (both inhibitory neuronal systems) need clarification as to why CB is unresponsive

Our reply: Theoretically, PV and CB interneurons has different types of ion channels and synaptic current kinetics, which together contributed to distinct neurodynamics and neural energy coding patterns across interneuron subtypes.

From a biological perspective, the fact that PV neurons exhibited greater changes in energy consumption under varying dopamine input suggested that PV interneurons was sensitive to dopamine modulation, which matched the experimental observation [10]. We have incorporated this interpretation into the **Discussion** section (paragraph 3). In future work, we plan to further investigate this phenomenon through additional simulations and experiments.

Minor Comment 3: plot Fig 7b,e,h in the same scale so that regional results can be appreciated

Our reply: We sincerely appreciate the reviewer's constructive suggestion. <u>We have modified Figure 7 in the revised manuscript.</u>

Reviewer #2

The quite well-written paper presents a biophysical model for a core circuit that includes the ventral tegmental area (VTA), the nucleus accumbens (NAc), and the medial prefrontal cortex, which is based on data from rodent experiments. The model is based on modified Hodgkin-Huxley equations for a total of 28 neurons, taking into account the specific properties of different relevant channels, and the Calcium dynamics, and also making specific assumptions about a highly simplified morphology of the neurons. The paper studies the behavior of this simulated circuit in major depressive disorder (MDD) by modeling variations changes in the neural dynamics that are dependent of changes of the dopamine concentration. The paper compares two ways of analysis, a classical analysis of the neural activity patterns and an analysis that investigates the electrical energy in different parts of the neural circuit. Both ways of analysis result partly in different dependencies of the behavior on the dopamine concentration, so that the neural energy analysis is proposed as an alternative way for analyzing the behavior of such neural structures, and maybe also a method to develop new clinical markers for system dysfunction.

Major points:

The paper presents a solid biophysical simulation study that contrasts neural activity patterns and an analysis in terms of the electrical energy of neural signals. Overall, the results seem convincing, and the approach is original. The study raises the following questions, which do not become entirely clear and that should be addressed prior to publication.

Comment 1: The paper proposes neural energy analysis as alternative way of analyzing disease related changes. While it is demonstrated that by simulation the model demonstrates specific changes in energy measures, which partly deviate from neural activity changes, the question emerges how such energy quantities can be measured directly in such circuits. This would make them most suitable as alternative clinical markers.

Our reply: This is a very good question. Regrettably, validating our neural energy simulation predictions remains challenging with current experimental techniques. The widely used *'Seahorse Cell Mito Stress Test'*, which assesses mitochondrial function to estimate bioenergetic energy expenditure in neurons, is typically conducted under *in vitro* conditions (e.g., brain slices). However, this experiment ignores the influence of synaptic projections between brain regions, making the *Seahorse Test* unable to measure the energy consumption of a certain neural circuit *in vivo*. Although brain imaging tools, such as BOLD-fMRI and FDG-PET, can measure brain energy metabolism *in vivo*, their low spatial and temporal resolution makes it difficult to apply them at the neural circuit level. Therefore, it would be difficult to find a appropriate way that we can use to validate our neural energy simulation results.

In addition, this limitation is a common challenge in computational neuroscience and extends beyond our current study, but we recognize the importance of this question and plan to collaborate with our wet lab partners to further investigate potential validation methods in future studies. <u>We have discussed these limitations in the final paragraph of the **Discussion** section</u>.

Comment 2: The modeling study is based on 9 repeated simulation of a model that contains at least 9 randomly chosen parameters. Is this number of repetitions really enough for obtaining stable results? This should be demonstrated or at least an argument should be given, why this is enough.

Our reply: We thank the reviewer for this question. In our previous study (in which we established the neurodynamical model for the VTA-NAc-mPFC neural circuit) [1], we have discussed the parameter settings and number of technical replicates for the model simulation. More specifically, in order to minimize the potential impact of random variables on

network dynamics, we set the random seeds (rng function in MATLAB) to control two key random variables (see **Table S11** in **Supplementary File 1**): 1) Dopamine concentration values, which varied randomly within predefined ranges (Low, Medium, High, Full); and 2) Network connection strengths. Through testing, we found that 9 technical replicates were sufficient for capturing stable trends in both neural network dynamics [1] and neural energy consumption patterns (this study and [9]). Additionally, due to computational constraints — our model involves 1,527 differential equation variables, requiring significant computational resources — we did not perform more replicates at this stage. In future studies, we aim to expand the number of neurons, random variables, and technical replicates to further refine our understanding of network dynamic.

Comment 3: The model makes very specific assumptions about a simplification of the anatomy of the underlying model and assumes in total only 28 neurons. Why was in particular this number of neurons chosen ? How sensitive are the results with respect to these assumptions. Ideally, some of these parameters or assumptions could be changed, showing that the key results hold even for a variation of some of these parameters.

Our reply: Similar to the *Comment 2*, we have also discussed the network structure and the parameter settings of the neuronal sample size in our previous study [1].

Our model mainly simulated the neural activities of two brain regions: the NAc and the mPFC (based on REF [11]). The NAc network consists of MSNs, PV, and CB interneurons, with physiological experiments demonstrating that MSNs dominate the neuronal population, accounting for ~95% of the total neuronal composition in this region [11]. In the mPFC region, the ratio of pyramidal neurons to interneurons is typically established at 4:1 [12].

However, in our model, the NAc region was simplified to 1 MSN, 1 PV interneuron, and 1 CB interneuron. This simplification arises because, if we strictly adhered to biological evidence, there should be at least 40 neurons in the NAc region (including 38 MSNs, 1 PV and 1 CB interneuron, if we assume a 1:1 ratio of PV to CB interneurons). Given that in our single MSN model, we have considered 14 different ion channel types with complex kinetic activity, resulting in a differential equation with 98 variables (based on REF [13], a detailed but highly complex model). Expanding the number of MSNs and incorporating all relevant factors would significantly increase the computation complexity, making calculations impractically long (our current model [14] takes over 20 hours per simulation, and we have spent more time to adjust model parameters and verify robustness). Therefore, we decided to simplify the network structures to 28 neurons for our VTA-NAc-mPFC microcircuit model.

To compensate for the reduced neuron count, we adjusted synaptic conductance values in accordance with neuron-type ratios in our model to align with biological evidence (see **Supplementary File 1**, and [14] for details). For example, MSNs in the NAc and pyramidal neurons in the mPFC are modulated by GABA (specifically $GABA_a$ receptors). Since there are only 2 interneurons (1 PV and 1 CB) releasing GABA onto MSNs in the NAc, and 5 interneurons (3 PV and 2 CB) releasing GABA onto pyramidal neurons in the mPFC, we adjusted the GABA conductance so that the total GABA conductance is approximately equal (0.3 for MSNs and 0.1 for pyramidal neurons, see **Supplementary File 1**). For estimating the conductance values, we referred to several computational and experimental studies [4]. Based on these appropriate biological assumptions and available experimental data [4, 7, 8, 12-14], we fine-tuned all parameters to their optimal values for the simulation. Our simulations has successfully replicated the dynamic characteristics of the VTA-NAc-mPFC neural circuit, including membrane potential, ion channel kinetics, synaptic dynamics, and field potentials [1], meaning that our simplification was biologically reasonable.

Minor points:

Comment 1: p.2, l. 21ff: What is the justification for the 28 neuron model and the relevant simplifications?

Our reply: Please see the response to *Reviewer #2*, **Comment 3**. For the modeling details, please see our previous study in REF [1].

Comment 2: * p. 7, last paragraph: I am wondering if the Pearson correlation coefficient is the right statistical measure for the relationship between the variables, since they might not be normally distributed. Otherwise, a Spearman correlation might be more appropriate. Also is seems that the correlations themselves apparently were not tested for significance.

Our reply: This is an important question to consider. We have carefully evaluated both Pearson (**Fig. 8**) and Spearman correlation coefficients (**Fig. R3**), which yielded different levels of statistical significance. However, we ultimately chose to use the **Pearson correlation coefficient** to measure the relationship between membrane potential and neural power.

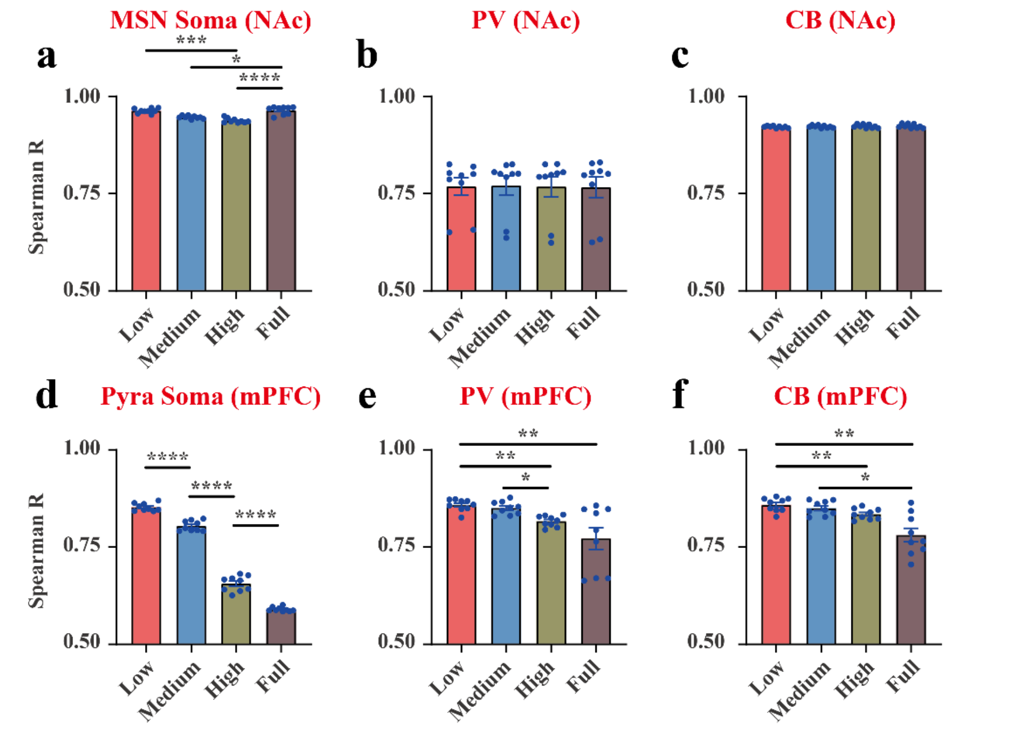

Figure R3 The Spearman correlation coefficients between the membrane potential curve and the actual neural power curve. n=9. *, p<0.05. **, p<0.01. ***, p<0.001. ****, p<0.0001.

The key reason for this choice was because the Spearman's correlation captures only the **monotonicity** of the relationship, whereas the Pearson's correlation quantifies the **numerical association** between membrane potential and neural power. . Since our study focuses on understanding the **quantitative relationship** between these two variables, Pearson's correlation is the more appropriate metric. More specifically, in both this manuscript and our previous research [1, 9], we have observed that when dopamine concentration is altered, certain neurons (e.g., MSN and CB interneurons in the mPFC regions) exhibit no significant changes in peak membrane potential, action potential frequency, or firing rate (Fig. 2, B-D, in REF [1]). However, their neural energy consumption patterns changes significantly (Fig. 3-4 in REF [9], and Fig. 3, Fig. 6f in the current manuscript). Measuring this relationship using the Spearman's correlation may ignore this numerical relationship.

Regarding the comment 'Also is seems that the correlations themselves apparently were not tested for significance.', we conducted statistical tests for significance when calculating Pearson's R (**Fig. 8**), using a **null hypothesis of** $r = 0$. The p values were all less than 1e-4, confirming that the correlations were all statistically significant. However, reporting individual p value in **Fig. 8** is challenging because each data point represents the Pearson correlation coefficient from a single replicated. Given that all Pearson's R in **Fig. 8** exceed 0.5 and the overall trend remains consistently stable, it is evident that these correlations are significantly nonzero. Therefore, we have opted not to report the individual p values in **Fig.8**.

Comment 3: * Are the 'Pyra neurons' pyramidal cells?

Our reply: Yes. We appreciate the reviewer's attention to detail. The use of "Pyra" to abbreviate pyramidal neurons is an legacy problem from our prior work in this research series [1, 9]. To maintain the consistency within this series, we have kept this abbreviation. We have added an Abbreviation section in the revised manuscript to summarize all abbreviations.

Comment 4: * Can there be an argument given why this assumption of uniform distributions of the different model parameters makes sense ? Same applies to the assumption of uniformly distributed connection strengths, which should have a strong influence on the network dynamics (omega parameters in formula S-12).

Our reply: In our manuscript, rather than fixing dopamine concentration and network connection strength to specific values, we simulate these variables using a uniform distribution within a defined range. This setting provides a more physiologically relevant representation of network dynamics and ensures that our simulation results remain robust under biologically plausible conditions. We chose not to use a normal distribution, as it could generate extreme values in these variables, potentially destroying the dynamical characteristics of our VTA-NAc-mPFC neural microcircuit.

Comment 5: * In the Supplement the derivation of the conductance of the compartment current using the Rall model (equation S-7) did not become clear to me. Either here a paper should be cited where this exact formula is derived, or an explicit explanation should be given, what exactly the underlying derivation is based on (length currents within the neurites; boundary conditions for the cable part etc.)

Our reply: The derivation and theoretical foundation of the Rall compartment neuronal model can be found in REF [15]. For a detailed explanation, please refer to page 64/440 of the online pdf https://neurophysics.ucsd.edu/courses/physics_171/Ermentrout_Therman.pdf. We have also cited this book and relevant publications in **Supplementary File 1**, page 3. Since the derivation of the Rall's model is not our original work, we only focus on its application to our model, which is presented in **Supplementary File 1 (Equations S7-S8)**.

Comment 6: * Supplement, section 3, last paragraph: Were these assumptions which conductances change with dopamine based on experimental data? On which one?

Our reply: Regarding dopamine-dependent kinetic changes, our parameter settings are primarily based on the modeling work of Durstewitz et al. [4], which incorporates detailed biological experiments. Further interpretation of this aspect can also be found in our previous modeling study [1].

Additional Editor Comments:

It is surprising, given that they consider neuronal energy, that the authors do not cite the classic paper by Attwell and Laughlin (2001) about energy budget in the brain (J. Cereb. Blood Flow Metabol. 2001). This paper should be mentioned and cited in the text. Also there is another, similar paper in topic, which should be cited in the text - Yu et al "Cerebral Cortex" 2022 ("A 3D atlas of functional....").

Our reply: We sincerely thank the editor for this suggestion. As this manuscript represents our second study on neural energy calculations in major depressive disorder (MDD), our previous work has already provided a detailed rationale for the importance and feasibility of applying neural energy approaches to MDD research. In this paper, we focused on the specific neural energy model [16], which is why we initially did not extensively cite or describe some foundational studies in the field. However, in response to the editor's suggestion, we have now incorporated the relevant citations in the revised manuscript (Attwell and Laughlin, 2001; Yu et al "Cerebral Cortex" 2022) to make our interpretation more clear.

The energy model in the paper (Eqs. 2 and 3) is somewhat abstract. In particular, it is not clear what is the biophysical sense of the total energy H_all? It should be explained that H_all relates to the ATP used by Na-K pumps that maintain concentration gradients of different ions. For this more biological approach, see J. Karbowski - J. Comput Neurosci. 2009 ("Thermodynamic constraints on neural dimensions .... "), and J. Karbowski - PLOS One 2012 ("Approximate invariance of metabolic energy...."). The authors should include these considerations in their paper.

Our reply: The energy model used in this study is an augmented version of the model in REF [16]. Unlike conventional approaches that estimate neural energy based on ATP consumption and the sodium-potassium pump, REF [16] directly computed the electrical power of the circuit based on the H-H equations, providing a different perspective on neural energy calculation. However, REF [16] was limited to the basic H-H model, which included only Na^+, K^+, and Cl^- ion channels, while excluding Ca^{2+} dynamics, complex Na^+ and K^+ ion channel subtypes, compartmental structures (soma, axon, dendrite structures), and chemical synapses. In our study, we enhanced the model [16] to enable a more comprehensive calculation of neural energy in our VTA-NAc-mPFC microcircuit.

Our H-H circuit model consists of **ion channels**, **synaptic currents**, **compartmental currents**, and **stimulus currents** (**Fig. 1e**). Among these, ion channel activity (ion currents) accounts for the largest proportion of neural energy expenditure due to the active transport of ions - we refer to this as the 'ionic neural energy' component in our paper (H_{Ions}). The remaining components (synaptic, compartment, and stimulus currents) are also integral to the H-H circuit and contribute to the overall power consumption, which we denote as $H_{Synapses}$, $H_{Compartment}$, $H_{Stimuli}$, respectively. The total energy consumption of the whole circuit is denoted as H_{all}. A detailed interpretation of this model can be found in **Supplementary File 1** and REFs [9, 16].

Currently, incorporating the Na-K pump mechanism into our neural energy calculations remains challenging, given that our model includes complex Na^+ and K^+ subtypes, Ca^{2+} dynamics and complex morphological structures (see **Supplementary File 1**). We have acknowledged the limitation in the final paragraph of the **Discussion** section and have added the editor's suggested citations. Moving forward, we plan to conduct additional experiments and simulations to further refine our neural energy calculations in the VTA-NAc-mPFC microcircuit and explore the consistency of different energy estimation methods.

In summary, we appreciate these very helpful comments and suggestions to improve our manuscript. We have responded to all the queries raised in the review, conducted additional experiments as requested and edited our manuscript accordingly. We sincerely hope that our revised manuscript is now suitable for publication in *PLoS Computational Biology*.

Thank you for your time and consideration.

Yours sincerely,

Yuanxi Li, PhD

Rubin Wang, PhD

Reference

1. Li Y, Zhang B, Pan X, Wang Y, Xu X, Wang R, et al. Dopamine-mediated major depressive disorder in the neural circuit of ventral tegmental area-nucleus accumbens-medial prefrontal cortex: from biological evidence to computational models. Frontiers in Cellular Neuroscience. 2022;16:923039.
2. Hodgkin AL, Huxley AF. A quantitative description of membrane current and its application to conduction and excitation in nerve. The Journal of physiology. 1952;117(4):500.
3. Destexhe A, Mainen ZF, Sejnowski TJ. Kinetic models of synaptic transmission. Methods in neuronal modeling. 1998;2:1-25.
4. Durstewitz D, Seamans JK, Sejnowski TJ. Dopamine-mediated stabilization of delay-period activity in a network model of prefrontal cortex. Journal of neurophysiology. 2000.
5. Onn S-P, Wang X-B, Lin M, Grace AA. Dopamine D1 and D4 receptor subtypes differentially modulate recurrent excitatory synapses in prefrontal cortical pyramidal neurons. Neuropsychopharmacology. 2006;31(2):318-38.
6. Perez MF, White FJ, Hu X-T. Dopamine D2 receptor modulation of K+ channel activity regulates excitability of nucleus accumbens neurons at different membrane potentials. Journal of neurophysiology. 2006;96(5):2217-28.
7. Yang CR, Seamans JK. Dopamine D1 receptor actions in layers V-VI rat prefrontal cortex neurons in vitro: modulation of dendritic-somatic signal integration. Journal of Neuroscience. 1996;16(5):1922-35.
8. Yang CR, Seamans JK, Gorelova N. Electrophysiological and morphological properties of layers V-VI principal pyramidal cells in rat prefrontal cortex in vitro. Journal of Neuroscience. 1996;16(5):1904-21.
9. Li Y, Zhang B, Liu Z, Wang R. Neural energy computations based on Hodgkin-Huxley models bridge abnormal neuronal activities and energy consumption patterns of major depressive disorder. Computers in Biology and Medicine. 2023;166:107500.
10. Nahar L, Grant CA, Hewett C, Cortes D, Reker AN, Kang S, et al. Regulation of Pv - specific interneurons in the medial prefrontal cortex and reward - seeking behaviors. Journal of neurochemistry. 2021;156(2):212-24.
11. Russo SJ, Nestler EJ. The brain reward circuitry in mood disorders. Nature reviews neuroscience. 2013;14(9):609-25.
12. Wang X-J, Tegnér J, Constantinidis C, Goldman-Rakic PS. Division of labor among distinct subtypes of inhibitory neurons in a cortical microcircuit of working memory. Proceedings of the National Academy of Sciences. 2004;101(5):1368-73.
13. Wolf JA, Moyer JT, Lazarewicz MT, Contreras D, Benoit-Marand M, O'Donnell P, et al. NMDA/AMPA ratio impacts state transitions and entrainment to oscillations in a computational model of the nucleus accumbens medium spiny projection neuron. Journal of Neuroscience. 2005;25(40):9080-95.
14. Li Y, Zhang B, Pan X, Wang Y, Xu X, Wang R, et al. Dopamine-mediated major depressive disorder in the neural circuit of ventral tegmental area-nucleus accumbens-medial prefrontal cortex: from biological evidence to computational models. Frontiers in cellular neuroscience. 2022;16.
15. Ermentrout B, Terman DH. Mathematical foundations of neuroscience: Springer; 2010.
16. Moujahid A, d'Anjou A, Torrealdea F, Torrealdea F. Energy and information in Hodgkin-Huxley neurons. Physical Review E—Statistical, Nonlinear, and Soft Matter Physics. 2011;83(3):031912.
